# Associations between climate variability, unemployment and suicide in Australia: a multicity study

**DOI:** 10.1186/s12888-015-0496-8

**Published:** 2015-05-12

**Authors:** Xin Qi, Wenbiao Hu, Andrew Page, Shilu Tong

**Affiliations:** School of Public Health, Xi’an Jiaotong University, Xi’an, Shaanxi 710061 China; School of Public Health and Social Work, Queensland University of Technology, Kelvin Grove, QLD 4059 Australia; School of Science and Health, University of Western Sydney, Building 24, Room 4.53D, Campbelltown Campus, Locked Bag, 1797, Penrith, NSW 2517 Australia

**Keywords:** Suicide, Australia, Cities, Climate, Unemployment

## Abstract

**Background:**

A number of studies have examined the associations of suicide with meteorological variables (MVs) and socioeconomic status but the results are inconsistent. This study assessed whether MVs and unemployment were associated with suicide in eight Australian capital cities.

**Methods:**

Data on suicide, population and unemployment rate (UER) between 1985 and 2005 were from the Australian Bureau of Statistics. MVs was provided by Australian Bureau of Meteorology. A generalized linear regression model with Poisson link was applied to explore the association of suicide with MVs and UER.

**Results:**

Temperature difference (ΔT, the difference in mean temperature between current month and previous one month) was positively associated with suicide in Sydney, Melbourne, Brisbane and Hobart. There was also a significant and positive association between UER and suicide in Sydney, Melbourne, Brisbane and Perth. MVs had more significant associations with violent suicide than that of non-violent suicide. There were no consistent associations between other MVs and suicide. A significant interaction between ΔT and UER on suicide was found in Sydney, Melbourne and Brisbane, such that increased temperature amplified the magnitude of the association between UER and suicide.

**Conclusions:**

ΔT and UER appeared to jointly influence the occurrence of suicide in Australian capital cities. This finding may have implications for developing effective suicide prevention strategies.

## Background

Suicide remains an important public health issue around the world [1]. Since the nineteenth century, the impact of socio-environmental factors on suicide have attracted much public attention, especially in the previous decades with the progress of global climate change and financial crisis [2–4]. Recently, a number of studies have explored this issue in different countries and found that some meteorological variables (MVs), e.g., temperature [5–13], rainfall [5, 12, 13], humidity and sunshine [11, 13], were associated with suicide risk. The seasonality of suicide were also examined [14–16]. However, all the previous studies were based on local environmental settings and the findings were inconsistent over different environmental settings. For example, a few studies indicated that increased temperature was accompanied with higher suicide risk [6, 7, 9, 13]; however, another study showed a suicide peak in winter with low temperature [5]. Some findings were contradictory even in the same area. For instance, Tsai and Cho [13] indicated that temperature was positively associated with suicide rate in Taiwan using a pure time-series analysis; while another study showed that the association was negative when comparing the regional differences of suicide rate in Taiwan [12]. These discrepancies may be due to the different regional focuses and different statistical methods being used [12].

In Australia, capital cities occupy less than 1 % of the total land area but contain 65 % of total population and account for approximately 60 % of suicides across the whole country [17–21]. Capital cities also represent a range of geographical, population size and climatic contexts, from the tropical north climate (e.g. Darwin) to the more temperate southern climate (e.g. Melbourne and Hobart). Thus the association of particular climate variable and suicide may differ across cities, and may affect other more proximal antecedents associated with suicide in populations. Some studies indicated that decreased rainfall and continued drought caused by rainfall deficiency were associated with higher suicide risk in New South Wales (NSW), especially in rural areas [22, 23]. Other studies showed that increased maximum temperature was accompanied with higher suicide rate over time and space in Queensland and Australia [24, 25]. However, each of these studies are based on only one state, thus the results from these studies are difficult to generalize to other areas. A few studies figured out that the associations between environmental factors and health status were modified by socioeconomic differences [26, 27]. Thus it is important to understand how MVs are associated with suicide rates and whether MVs have interactive effects on socioeconomic factors (e.g. unemployment) and suicide in various cities over time, in order to understand how the epidemiology of suicide in Australia differs geographically and to inform suicide prevention programmes locally.

Most suicides occurred in capital cities and climate patterns within each city are relatively homogeneous compared to rural and remote areas with large geographical size, sparse population and suicide cases, and varied climate factors. Thus this study aimed to explore the pattern of suicide in Australian capital cities, and to assess the association of suicide with MVs and unemployment rate in these cities, and extent to which MVs modified associations between unemployment and suicide.

## Methods

### Data sources

**Fig. 1:**
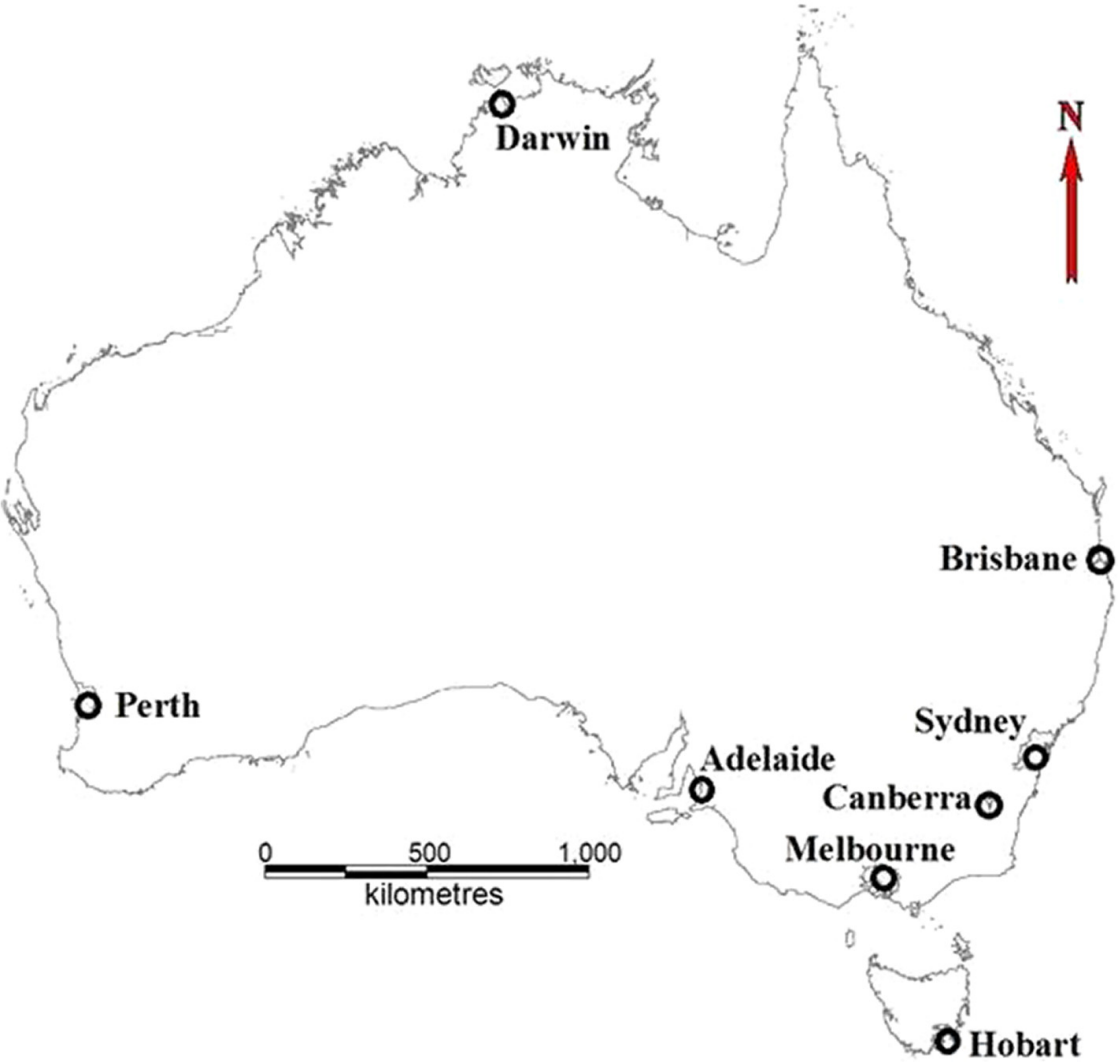
The locations of capital cities in Australia

Suicide data (1985 to 2005), including sex, age, International Classification of Disease (ICD) Code relating to suicide or self-inflicted injury (ICD 9:950.0-952.9 for non-violent suicide and 953.0-959.9 for violent suicide; ICD 10: ×60.0- × 69.9 for non-violent suicide and × 70.0- × 84.9 for violent suicide), suicide date, country of birth and statistical local area (SLA) code, were provided by Australian Bureau of Statistics (ABS). As around 5 % of total selected data has only “year” and “month” of suicide but no information of accurate “day” (especially in 2004 due to the quality of original data), and some cities have a relatively small size of population and number of suicide (e.g., Darwin and Hobart), we used monthly data in this study to assure covering relatively recent data (2004 and 2005) and cities with more diverse climate. Suicide data after 2005 were not included because the related procedure for accessing recent ABS data is currently under review. According to the definition from ABS, we used eight Statistical Divisions (SDs) to represent the metropolitan areas of eight Australian capital cities in different climate zones: one in tropical climate zone (Darwin), one in sub-tropical climate zone (Brisbane) and other six in temperate climate zone (Sydney, Melbourne, Adelaide, Perth, Hobart, and Canberra). Each SD was composed of a certain number of SLAs and suicide data at the SLA level were aggregated into SD level. The locations of each city were shown in Fig. 1. The map in Fig. 1 was generated by MapInfo 10.5 using the geographical boundary from ABS [28]. Census population data (SD level, 1986, 1991, 1996, 2000, 2005) by age and sex and monthly unemployment rate (UER, %, total and by sex, seasonally-adjusted), were also obtained from ABS. We interpolated the population size by sex and age groups for each year using census population data. The institutional ethics approval was granted by the Human Research Ethics Committee, Queensland University of Technology.

Monthly meteorological variables (MVs), including rainfall (mm), relative humidity (%), maximum and minimum temperature (Tmax and Tmin, °C), sunshine hours (daily average of each month), were supplied by the Australian Bureau of Meteorology. We examined the monitoring stations within the boundaries of all metropolitan areas of eight capital cities, selected the stations which had valid records covering the whole study period, calculated the mean values of station records in each city, and applied them in the data analyses. The monthly mean temperature (T-mean, °C) was calculated by using the mean value of Tmax and Tmin. As temperature has been found having more significant associations with suicide in published literature than other MVs, we also calculated the temperature difference (ΔT, the difference in mean temperature between current month and previous one month, °C; above 0 °C for increase and below 0 °C for decrease) for following data analyses.

### Data analysis

A series of statistical methods were applied to investigate associations of MVs and UER with suicide rate. Seasonal differences of suicide in each city were explored using the mean mortality of 12-month cycles. Monthly age-adjusted mortality rate (per 100,000) by sex in each city was calculated. Spearman correlation analysis was applied to assess multicollinearity of independent variables, particularly the temperature indexes. The variables with high multicollinearity (correlation ≥ |0.80|) were included in separated models. A generalized linear model (GLM) with Poisson link was used to investigate whether MVs and the UER were associated with suicide rates across different cities. Then we selected the MVs having the strongest associations with suicide and tested the interaction of MVs on the association between UER and suicide. Monthly rainfall, temperature, ΔT, humidity, sunshine hours and UER were used as independent variables in GLM. UER were added in GLM with different lags (1 to 6 months). Population in each city (total and by sex) with logarithm transfer was used as an offset. Seasonality (Darwin: September to February for wet season; March to August for dry season; other cities: September to November for spring; December to February for summer, March to May for autumn; June to August for winter) was also adjusted as categorical independent variables. All the MVs were seasonally adjusted. The minimum value of Akaike information criterion (AIC) was applied to select the most suitable model. Autocorrelations of residuals for each model were also checked. All the analyses were conducted using the software package SPSS 21.0 [29].

## Results

### Suicide, climate and unemployment in Australian capital cities

Twenty-eight thousand five hundred one suicide cases (21,999 males and 6,502 females) from eight cities between 1985 and 2005 were included in this study. Sydney and Melbourne had the largest number of suicide case (16,665 for both) and accounted for 58.5 % of total suicides, followed by Brisbane, Perth and Adelaide. Darwin, Hobart and Canberra had the smallest number of suicide cases (1,662 for all three cities and 5.8 % of total suicides). Table 1 shows the distribution of suicide rates, meteorological variables (MVs) and unemployment status based on the mean monthly total. Sydney, Melbourne and Canberra had the lowest suicide rates while Darwin had the highest rates. Darwin also had the highest mean temperature, rainfall and sunshine among all selected cities due to its tropical climate. Hobart had the highest UER, followed by Adelaide and Brisbane; while Canberra had the lowest UER.

**Table 1:** Summary statistics for meteorological variables, unemployment rate and suicide rates in Australian capital cities (1985–2005, mean of monthly total)

Variables	Cities	Mean	SD	Min		Percentiles		Max
					25	50	75	
Mortality rate (M, F, per 100,000)	Sydney	0.96 (1.51, 0.44)	0.22 (0.38, 0.19)	0.43 (0.71, 0.05)	0.81 (1.22, 0.30)	0.94 (1.47, 0.42)	1.10 (1.74, 0.59)	1.76 (2.94, 1.07)
	Melbourne	0.97 (1.50, 0.47)	0.22 (0.38, 0.18)	0.33 (0.47, 0.05)	0.83 (1.23, 0.35)	0.96 (1.50, 0.47)	1.12 (1.75, 0.59)	1.56 (2.41, 0.96)
	Brisbane	1.15 (1.84, 0.49)	0.35 (0.58, 0.28)	0.27 (0.41, 0.00)	0.90 (1.38, 0.29)	1.15 (1.78, 0.44)	1.40 (2.28, 0.65)	2.28 (3.83, 1.63)
	Adelaide	1.03 (1.63, 0.46)	0.34 (0.59, 0.31)	0.37 (0.39, 0.00)	0.80 (1.21,0.20)	1.00 (1.60, 0.39)	1.24 (2.02, 0.59)	2.00 (3.41, 1.53)
	Perth	1.06 (1.69, 0.46)	0.33 (0.57, 0.29)	0.34 (0.41, 0.00)	0.82 (1.25, 0.27)	1.04 (1.60, 0.44)	1.29 (2.13, 0.63)	1.99 (3.28, 1.32)
	Hobart	1.27 (2.07, 0.51)	0.82 (1.43, 0.78)	0.00 (0.00, 0.00)	0.53 (1.08, 0.00)	1.08 (2.16, 0.00)	1.64 (3.24, 1.03)	3.95 (7.56, 4.06)
	Darwin	1.41 (2.34, 0.40)	1.22 (2.14, 0.92)	0.00 (0.00, 0.00)	0.00 (0.00, 0.00)	1.13 (2.19, 0.00)	2.01 (3.50, 0.00)	5.02 (8.78, 5.73)
	Canberra	0.97 (1.52, 0.42)	0.59 (1.03, 0.50)	0.00 (0.00, 0.00)	0.61 (0.72, 0.00)	0.92 (1.36, 0.00)	1.34 (2.03, 0.71)	3.23 (5.34, 1.98)
Rainfall (mm)	Sydney	91.4	80.18	0.7	39.9	70.0	124.5	560.6
	Melbourne	62.5	30.98	3.3	40.3	58.5	83.5	166.9
	Brisbane	91.4	81.43	0.8	39.9	70.1	122.2	609.8
	Adelaide	55.8	40.82	0.0	19.4	50.0	84.5	193.7
	Perth	70.1	65.51	0.0	12.8	52.7	115.3	266.8
	Hobart	57.2	34.78	2.6	33.0	50.8	74.9	255.2
	Darwin	137.8	164.90	0.0	1.7	71.5	234.9	841.5
	Canberra	54.6	40.10	0.6	24.0	46.8	73.9	248.2
Humidity ( %)	Sydney	63.5	6.97	41.6	58.8	64.1	68.3	79.4
	Melbourne	64.9	7.15	46.7	59.3	64.4	70.5	79.8
	Brisbane	63.4	5.57	43.7	59.9	63.5	67.2	78.6
	Adelaide	56.6	11.05	35.9	47.5	54.5	65.9	77.5
	Perth	59.8	8.93	43.8	51.8	59.6	67.8	77.8
	Hobart	61.9	6.59	45.5	57.5	61.8	66.5	77.0
	Darwin	62.0	11.21	37	52	61.5	72.5	85.0
	Canberra	60.1	9.37	38	53.1	60.3	67.0	79.0
T (ΔT,°C)	Sydney	17.19 (0.01)	3.87 (2.26)	10.36 (−4.82)	13.89 (−1.76)	17.43 (0.20)	20.66 (1.98)	24.41 (4.29)
	Melbourne	14.84 (0.01)	3.70 (2.24)	8.58 (−6.09)	11.41 (−1.81)	14.81 (0.23)	18.15 (1.69)	23.31 (5.23)
	Brisbane	20.36 (0.01)	3.67 (2.11)	13.79 (−4.45)	17.09 (−1.84)	20.86 (0.05)	23.55 (1.83)	26.50 (3.93)
	Adelaide	16.83 (0.00)	4.29 (2.62)	9.49 (−8.21)	12.90 (−1.97)	16.69 (0.38)	20.58 (1.98)	26.83 (7.15)
	Perth	18.37 (−0.01)	4.01 (2.40)	11.66 (−5.36)	14.60 (−1.67)	18.05 (0.05)	22.10 (1.73)	27.81 (6.03)
	Hobart	12.97 (0.01)	3.14 (1.91)	7.28 (−4.28)	10.05 (−1.59)	12.93 (0.30)	15.67 (1.49)	19.63 (3.95)
	Darwin	27.52 (0.00)	1.83 (1.39)	22.45 (−4.10)	26.28 (−0.84)	28.03 (−0.10)	28.84 (0.81)	30.23 (3.75)
	Canberra	13.39 (0.01)	5.24 (3.00)	4.65 (−6.25)	8.91 (−2.56)	13.45 (0.30)	18.14 (2.70)	22.80 (5.80)
Sunshine hours (daily average)	Sydney	7.1	1.21	3.7	6.4	7.1	7.9	10.4
	Melbourne	6.1	1.69	2.7	4.8	6.2	7.4	9.9
	Brisbane	8.1	1.22	4.4	7.3	8.2	8.8	10.6
	Adelaide	7.6	2.08	3.3	5.8	7.8	9.3	11.9
	Perth	8.5	2.22	4.0	6.8	8.2	10.5	12.6
	Hobart	6.5	1.52	3.2	5.3	6.4	7.7	10.6
	Darwin	8.6	1.89	3.0	7.4	9.2	10.1	11.0
	Canberra	7.7	1.69	3.6	6.3	7.8	9.0	11.4
UER (M, F, %)	Sydney	7.4 (7.5,7.3)	1.7 (1.9,1.5)	5.0 (5.0, 4.8)	5.9 (5.9, 5.9)	7.3 (7.4, 7.2)	8.6 (8.5, 8.8)	11.0 (11.9, 9.8)
	Melbourne	7.5 (7.4, 7.7)	2.1 (2.4,1.8)	4.7 (4.0, 5.4)	5.8 (5.6, 5.9)	6.7 (6.4, 7.6)	8.9 (9.1, 8.8)	12.5 (12.8, 12.3)
	Brisbane	8.4 (8.3, 8.5)	1.6 (1.8,1.4)	4.7 (4.3, 5.1)	7.4 (7.2, 7.7)	8.6 (8.6, 8.6)	9.7 (9.5, 9.8)	10.8 (11.3, 10.7)
	Adelaide	8.5 (8.9, 8.0)	1.7 (2.0,1.5)	4.8 (5.2, 4.1)	7.2 (7.7, 6.6)	8.7 (8.7, 8.5)	9.6 (10.3, 9.1)	12.0 (13.2, 10.5)
	Perth	7.3 (7.4, 7.3)	1.6 (1.8,1.5)	4.1 (3.8, 4.4)	6.1 (6.5, 6.0)	7.3 (7.3, 7.3)	8.0 (7.9, 8.5)	11.1 (11.6, 10.9)
	Hobart	9.4 (10.0, 8.7)	1.6 (1.9,1.5)	5.8 (6.1, 5.5)	8.7 (8.8, 7.7)	9.3 (9.8, 9.1)	10.6 (11.5, 9.9)	13.0 (14.1, 15.4)
	Darwin	6.6 (6.7. 6.4)	1.6 (1.8,1.7)	3.7 (3.0, 2.7)	5.3 (5.3, 5.0)	6.6 (6.5, 6.4)	7.5 (7.6, 7.5)	11.2 (11.9, 10.9)
	Canberra	5.7 (6.0, 5.5)	1.4 (1.5,1.5)	3.0 (3.0, 2.7)	4.6 (4.9, 4.2)	5.4 (5.6, 5.4)	7.0 (7.5, 6.7)	8.60 (8.8, 9.2)

Note: SD: standard deviation; Min: minimum, Max: maximum; M: male; F: female; T: temperature; ΔT: temperature increase; UER: unemployment rate

### Seasonality of suicide

**Fig. 2:**
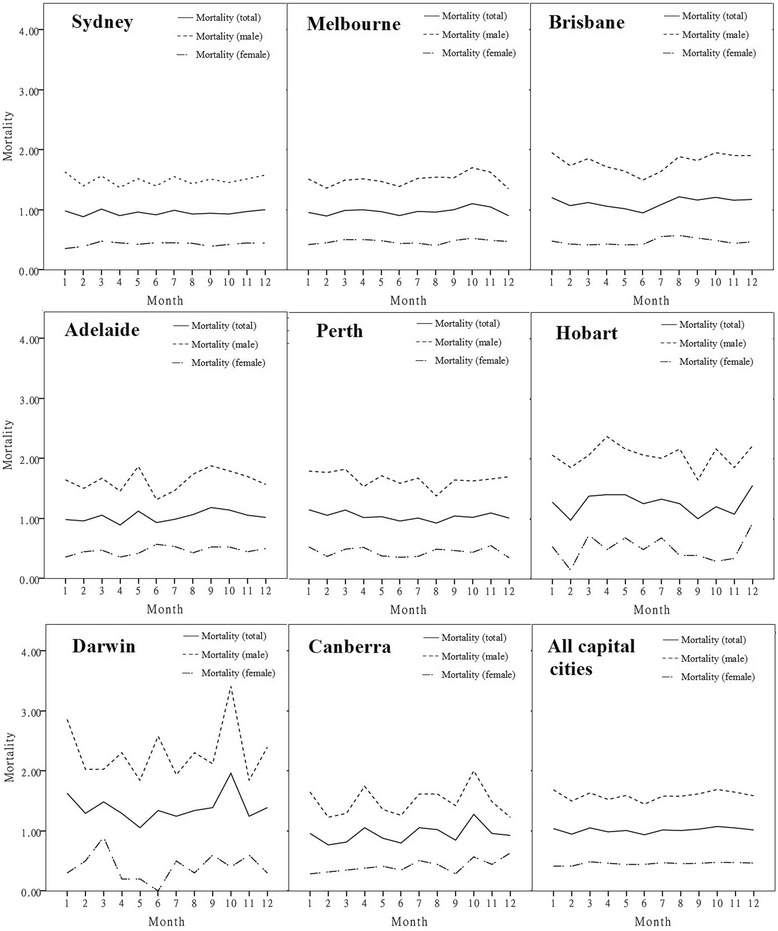
Monthly suicide rate by city (total and by sex)

Figure 2 demonstrates various seasonal trend of suicide rates in different cities. There was no obvious seasonal variation of suicide rates in Sydney, while Melbourne had a small peak of suicide (in males) in spring. Brisbane experienced the lowest suicide rates in June (winter), and high but stable suicide rate from spring to summer; which was similar to Perth. In Adelaide, two peaks were observed in May and October (predominantly in males). A sharp peak (predominantly in males) of suicide in October was also found in Darwin and Canberra.

### Association of suicide with climate and unemployment

The results of GLM modeling show that ΔT was positively associated with the suicide in Sydney (for males and total rates), Melbourne (in males), Brisbane (in males and total rates), Hobart (in females and total rates) and Canberra (in females) (Table 2). Temperature was only positively (marginally significant) associated with total and male suicide in Darwin. A significant association between humidity and suicide was also found among males in Darwin. Rainfall had a negative association with total and female suicide in Melbourne. No significant associations were discovered between MVs and suicide in Adelaide and Perth. Other MVs were also tested in the models. The associations between maximum temperature and suicide rates were similar as that of mean temperature, while minimum temperature appeared to be less significant. There was a positive association between UER (1 month lag) and suicide in Sydney (total and by sex), Melbourne (total and male), Brisbane (total and by sex) and Perth (total and male). However, this association was not significant in other cities. The results of the pooled data indicate that both ΔT and UER were positively associated with suicide. The associations between adjacent monthly changes of other MVs (besides temperature) and suicide were examined, with less significant associations discovered than that of temperature.

**Table 2:** The association between meteorological variables, unemployment rate and suicide in Australian capital cities (total and by sex)

City	Parameter Estimates	All				Male				Female			
		RR	95 % CI		P-value	RR	95 % CI		P-value	RR	95 % CI		P-value
Sydney	Rainfall (100 mm)	1.00	0.97	1.04	0.921	0.99	0.95	1.03	0.700	1.02	0.95	1.08	0.632
	Humidity (%)	1.00	1.00	1.01	0.635	1.00	0.99	1.01	0.754	1.00	0.99	1.01	0.858
	Temperature (°C)	1.01	0.99	1.02	0.356	1.00	0.99	1.02	0.948	1.02	1.00	1.05	0.097
	ΔT (°C)	1.03	1.01	1.04	0.001	1.02	1.01	1.04	0.010	1.03	1.00	1.06	0.089
	Sunshine hours	0.98	0.95	1.01	0.229	0.98	0.94	1.02	0.308	0.94	0.87	1.01	0.092
	UER (%)	1.05	1.03	1.06	0.001	1.04	1.02	1.05	0.001	1.09	1.06	1.12	0.001
Melbourne	Rainfall (100 mm)	0.91	0.82	1.00	0.034	0.93	0.83	1.03	0.151	0.84	0.67	1.01	0.049
	Humidity (%)	1.01	1.00	1.01	0.130	1.01	1.00	1.01	0.121	1.00	0.99	1.01	0.892
	Temperature (°C)	1.00	0.98	1.02	0.998	0.99	0.97	1.01	0.585	1.01	0.98	1.05	0.479
	ΔT (°C)	1.02	1.00	1.03	0.097	1.02	1.00	1.04	0.033	0.99	0.95	1.02	0.536
	Sunshine hours	1.02	0.99	1.06	0.202	1.04	1.00	1.08	0.043	0.96	0.89	1.03	0.286
	UER (%)	1.02	1.01	1.03	0.001	1.02	1.01	1.03	0.001	1.01	0.98	1.03	0.580
Brisbane	Rainfall (100 mm)	0.97	0.92	1.03	0.319	0.97	0.91	1.03	0.279	0.97	0.86	1.08	0.589
	Humidity (%)	1.00	0.99	1.01	0.716	1.00	0.99	1.01	0.893	0.99	0.97	1.02	0.560
	Temperature (°C)	1.01	0.99	1.03	0.458	1.01	0.99	1.04	0.364	0.99	0.95	1.04	0.798
	ΔT (°C)	1.03	1.01	1.06	0.010	1.04	1.01	1.07	0.015	1.03	0.97	1.08	0.315
	Sunshine hours	0.97	0.92	1.01	0.144	0.96	0.91	1.01	0.152	0.98	0.88	1.08	0.627
	UER (%)	1.05	1.03	1.07	0.001	1.04	1.02	1.06	0.001	1.09	1.04	1.13	0.001
Adelaide	Rainfall (100 mm)	1.06	0.90	1.22	0.482	1.09	0.90	1.27	0.385	1.00	0.66	1.33	0.976
	Humidity (%)	1.00	0.99	1.01	0.712	1.00	0.98	1.01	0.932	1.01	0.99	1.04	0.340
	Temperature (°C)	0.99	0.96	1.02	0.391	0.98	0.95	1.01	0.158	1.03	0.97	1.08	0.401
	ΔT (°C)	0.98	0.96	1.01	0.171	0.99	0.96	1.02	0.490	0.96	0.91	1.01	0.126
	Sunshine hours	1.01	0.96	1.07	0.634	1.02	0.96	1.08	0.484	0.99	0.87	1.10	0.830
	UER (%)	0.99	0.96	1.01	0.223	0.98	0.96	1.01	0.165	1.00	0.95	1.05	0.951
Perth	Rainfall (100 mm)	1.10	0.97	1.22	0.152	1.09	0.95	1.23	0.227	1.05	0.78	1.32	0.722
	Humidity (%)	0.99	0.98	1.00	0.158	0.99	0.98	1.00	0.224	0.99	0.96	1.02	0.422
	Temperature (°C)	1.00	0.97	1.03	0.979	1.00	0.97	1.03	0.943	1.00	0.94	1.05	0.941
	ΔT (°C)	1.02	0.99	1.04	0.259	1.01	0.98	1.04	0.358	1.02	0.96	1.08	0.516
	Sunshine hours	1.02	0.97	1.07	0.442	1.00	0.95	1.05	0.988	1.07	0.96	1.17	0.247
	UER (%)	1.05	1.02	1.07	0.001	1.04	1.02	1.06	0.001	1.04	0.99	1.09	0.131
Hobart	Rainfall (100 mm)	1.09	0.85	1.34	0.471	1.04	0.76	1.32	0.785	1.30	0.80	1.79	0.303
	Humidity (%)	1.01	0.99	1.04	0.321	1.01	0.98	1.03	0.625	1.05	0.99	1.11	0.091
	Temperature (°C)	0.96	0.89	1.03	0.229	0.97	0.90	1.05	0.462	0.91	0.76	1.07	0.247
	ΔT (°C)	1.08	1.01	1.15	0.032	1.04	0.97	1.12	0.301	1.25	1.09	1.40	0.005
	Sunshine hours	1.06	0.92	1.20	0.403	1.05	0.90	1.21	0.506	1.15	0.84	1.46	0.381
	UER (%)	1.00	0.95	1.06	0.923	1.02	0.97	1.07	0.375	0.91	0.78	1.03	0.129
Darwin	Rainfall (100 mm)	1.06	0.90	1.22	0.458	1.09	0.92	1.26	0.326	0.94	0.41	1.47	0.812
	Humidity (%)	0.98	0.95	1.01	0.223	0.97	0.93	1.00	0.043	1.11	1.00	1.21	0.052
	Temperature (°C)	1.16	1.02	1.29	0.031	1.18	1.03	1.32	0.025	0.93	0.55	1.31	0.701
	ΔT (°C)	1.07	0.95	1.18	0.282	1.08	0.96	1.21	0.194	0.91	0.59	1.24	0.586
	Sunshine hours	0.99	0.80	1.17	0.876	0.94	0.74	1.13	0.517	1.51	0.95	2.07	0.152
	UER (%)	0.95	0.88	1.03	0.194	0.96	0.89	1.03	0.235	0.63	0.42	0.84	0.001
Canberra	Rainfall (100 mm)	1.12	0.88	1.36	0.359	1.21	0.94	1.48	0.173	0.84	0.29	1.39	0.532
	Humidity (%)	1.00	0.98	1.02	0.955	1.00	0.98	1.02	0.986	1.01	0.96	1.05	0.727
	Temperature (°C)	0.97	0.94	1.01	0.181	0.97	0.93	1.02	0.212	0.98	0.90	1.06	0.630
	ΔT (°C)	1.02	0.98	1.07	0.319	1.01	0.95	1.06	0.839	1.09	0.99	1.19	0.074
	Sunshine hours	1.07	0.95	1.19	0.259	1.08	0.95	1.22	0.246	1.05	0.80	1.29	0.709
	UER (%)	1.03	0.98	1.08	0.277	1.04	0.98	1.10	0.180	1.03	0.93	1.14	0.552
All eight cities	Rainfall (100 mm)	0.99	0.97	1.02	0.597	0.99	0.96	1.02	0.507	1.00	0.95	1.05	0.926
	Humidity (%)	1.00	1.00	1.01	0.196	1.00	1.00	1.01	0.334	1.00	0.99	1.01	0.670
	Temperature (°C)	1.00	0.99	1.01	0.644	1.00	0.99	1.01	0.882	1.00	0.99	1.02	0.694
	ΔT (°C)	1.02	1.01	1.03	0.001	1.02	1.01	1.03	0.001	1.01	1.00	1.03	0.135
	Sunshine hours	1.00	0.99	1.02	0.822	1.00	0.98	1.02	0.975	1.00	0.96	1.03	0.936
	UER (%)	1.03	1.02	1.03	0.001	1.02	1.02	1.03	0.001	1.03	1.02	1.05	0.001

Note: ΔT (temperature increase), UER (unemployment rate); Seasonality was adjusted

Table 3 explores the associations of MVs and UER with suicide by methods. There was no significant association of rainfall and humidity with either violent or non-violent suicide in all cities. No significant association of suicide with MVs and UER was discovered in Adelaide, Hobart, Darwin and Canberra. MVs (e.g., ΔT) were only associated with violent suicide. However, non-violent suicide had higher relative risk (RR) in associating with UER than that of violent suicide in Sydney, Melbourne, Brisbane, Perth and all cities together.

**Table 3:** The association between meteorological variables, unemployment rate and suicide in Australian capital cities (by suicide methods)

City	Parameter Estimates	Violent				Non-violent			
		RR	95 % CI		P-value	RR	95 % CI		P-value
Sydney	Temperature (°C)	1.01	0.99	1.02	0.368	1.01	0.98	1.03	0.580
	ΔT (°C)	1.03	1.01	1.06	0.002	1.01	0.98	1.04	0.541
	Sunshine hours	0.99	0.95	1.03	0.642	0.96	0.90	1.02	0.160
	UER (%)	1.03	1.02	1.05	0.001	1.08	1.06	1.10	0.001
Melbourne	Temperature (°C)	0.99	0.96	1.01	0.185	1.00	0.98	1.03	0.769
	ΔT (°C)	1.02	1.00	1.04	0.105	1.00	0.97	1.03	0.860
	Sunshine hours	1.06	1.02	1.11	0.010	1.02	0.96	1.08	0.517
	UER (%)	1.01	1.00	1.02	0.124	1.03	1.01	1.05	0.002
Brisbane	Temperature (°C)	1.03	1.01	1.06	0.021	0.97	0.93	1.01	0.100
	ΔT (°C)	1.04	1.01	1.07	0.019	1.02	0.98	1.06	0.301
	Sunshine hours	0.95	0.90	1.01	0.106	0.99	0.91	1.06	0.710
	UER (%)	1.03	1.00	1.06	0.022	1.08	1.05	1.11	0.001
Perth	Temperature (°C)	1.01	0.97	1.04	0.670	0.99	0.95	1.03	0.621
	ΔT (°C)	1.00	0.97	1.04	0.924	1.03	0.99	1.07	0.123
	Sunshine hours	1.02	0.96	1.09	0.491	1.02	0.95	1.10	0.586
	UER (%)	1.01	0.98	1.04	0.684	1.10	1.06	1.13	0.001
All eight cities	Temperature (°C)	1.01	1.00	1.01	0.284	1.00	0.99	1.01	0.678
	ΔT (°C)	1.02	1.01	1.03	0.001	1.01	0.99	1.02	0.322
	Sunshine hours	1.00	0.97	1.02	0.647	1.01	0.99	1.04	0.378
	UER (%)	1.01	1.00	1.02	0.011	1.05	1.04	1.06	0.001

Note: ΔT (temperature increase), UER (unemployment rate); Seasonality was adjusted

### The interaction of ΔT and unemployment on suicide

As ΔT and unemployment were two key predictors of suicide, the interactive effects of these two variables on suicide were also examined by dividing both of UER (1 month lag) and ΔT into two levels (lower or higher than the median value in each city in the whole study period), respectively (Tables 4 and 5), using months with low ΔT and low UER as reference group. The Chi-square test indicated that most cities had significant overall effects except for Hobart (Table 4). In Table 5, months with high ΔT and high UER and months with low ΔT but high UER had higher suicide in Sydney, Melbourne, Brisbane and all cities together. Months with low ΔT but high UER had higher suicide risk only in Brisbane. In Canberra, suicide risk was only high in months with both high ΔT and UER. Only Adelaide had lower suicide risk in months of high ΔT and low UER (female) and months of low ΔT and high UER (all). There are no significant association in Perth, Hobart and Darwin.

**Table 4:** Chi square test of the interaction of temperature on unemployment rate

Cities	Chi-square	P-value
Sydney	9.815	0.020
Melbourne	31.649	<0.001
Brisbane	35.984	<0.001
Adelaide	42.074	<0.001
Perth	10.007	0.019
Hobart	1.358	0.715
Darwin	15.747	0.001
Canberra	10.221	0.017
All eight cities	17.402	0.001

**Table 5:** The interaction of temperature on unemployment rate on suicide

City	Parameter Estimates	All				Male				Female			
		RR	95 % CI		P-value	RR	95 % CI		P-value	RR	95 % CI		P-value
Sydney	High ΔT*High UER	1.22	1.14	1.29	0.001	1.20	1.12	1.29	0.001	1.27	1.11	1.43	0.003
	Low ΔT* High UER	1.13	1.07	1.19	0.001	1.11	1.04	1.18	0.005	1.26	1.14	1.39	0.001
	High ΔT*Low UER	1.01	0.93	1.09	0.855	0.99	0.90	1.18	0.891	1.00	0.83	1.16	0.982
	Low ΔT* Low UER	1				1				1			
Melbourne	High ΔT*High UER	1.14	1.06	1.22	0.001	1.15	1.05	1.24	0.005	1.18	1.02	1.35	0.044
	Low ΔT* High UER	1.06	1.00	1.13	0.061	1.08	1.00	1.15	0.049	1.14	1.00	1.27	0.051
	High ΔT*Low UER	0.97	0.88	1.05	0.413	0.99	0.89	1.08	0.790	1.02	0.86	1.19	0.781
	Low ΔT* Low UER	1				1				1			
Brisbane	High ΔT*High UER	1.32	1.21	1.43	0.001	1.34	1.22	1.46	0.001	1.33	1.08	1.58	0.023
	Low ΔT* High UER	1.09	1.00	1.18	0.056	1.05	0.94	1.15	0.374	1.29	1.10	1.48	0.010
	High ΔT*Low UER	1.18	1.07	1.28	0.003	1.17	1.04	1.29	0.014	1.21	0.97	1.44	0.120
	Low ΔT* Low UER	1				1				1			
Adelaide	High ΔT*High UER	0.92	0.79	1.06	0.257	0.92	0.77	1.08	0.340	0.86	0.59	1.13	0.291
	Low ΔT* High UER	0.88	0.77	0.99	0.023	0.91	0.78	1.03	0.135	0.82	0.60	1.05	0.085
	High ΔT*Low UER	0.89	0.76	1.03	0.110	0.97	0.82	1.13	0.729	0.73	0.44	1.02	0.030
	Low ΔT* Low UER	1				1				1			
Perth	High ΔT*High UER	1.11	0.97	1.25	0.129	1.08	0.93	1.24	0.315	1.13	0.84	1.42	0.423
	Low ΔT* High UER	1.04	0.94	1.15	0.385	1.06	0.95	1.17	0.294	1.01	0.79	1.23	0.903
	High ΔT*Low UER	0.94	0.81	1.08	0.405	0.94	0.79	1.10	0.475	0.98	0.68	1.28	0.909
	Low ΔT* Low UER	1				1				1			
Hobart	High ΔT*High UER	1.12	0.86	1.38	0.390	0.96	0.66	1.26	0.790	1.26	0.71	1.81	0.407
	Low ΔT* High UER	0.93	0.70	1.16	0.527	1.01	0.76	1.26	0.950	0.55	0.02	1.08	0.026
	High ΔT*Low UER	0.99	0.72	1.26	0.938	1.06	0.76	1.35	0.700	1.10	0.51	1.70	0.753
	Low ΔT* Low UER	1				1				1			
Darwin	High ΔT*High UER	0.85	0.45	1.24	0.414	1.11	0.72	1.50	0.603	0.90	−0.05	1.84	0.822
	Low ΔT* High UER	0.90	0.57	1.23	0.533	1.27	0.93	1.60	0.166	0.71	−0.22	1.65	0.479
	High ΔT*Low UER	1.13	0.81	1.45	0.463	1.44	1.06	1.82	0.062	0.93	0.06	1.80	0.874
	Low ΔT* Low UER	1				1				1			
Canberra	High ΔT*High UER	1.34	1.05	1.62	0.049	1.32	0.99	1.65	0.104	1.42	0.82	2.01	0.253
	Low ΔT* High UER	1.09	0.87	1.31	0.444	1.11	0.86	1.36	0.418	1.06	0.60	1.53	0.793
	High ΔT*Low UER	1.18	0.88	1.48	0.279	1.25	0.91	1.59	0.198	1.05	0.45	1.65	0.875
	Low ΔT* Low UER	1				1				1			
All eight cities	High ΔT*High UER	1.15	1.11	1.19	0.001	1.15	1.10	1.19	0.001	1.16	1.07	1.24	0.001
	Low ΔT* High UER	1.05	1.02	1.09	0.002	1.06	1.02	1.10	0.003	1.10	1.03	1.17	0.005
	High ΔT*Low UER	1.00	0.96	1.04	0.962	1.02	0.97	1.07	0.477	0.99	0.90	1.08	0.883
	Low ΔT* Low UER	1				1				1			

Note: UER (unemployment rate); ΔT (temperature increase). Seasonality, rainfall, temperature, humidity and sunshine were adjusted. The median ΔT and UER of the whole study period in each city were applied to identify high or low ΔT and UER in each city

## Discussion

This study examined the association of suicide with meteorological variables (MVs) and unemployment rate (UER) in eight Australian capital cities. There was a relatively more significant association across all cities between ΔT and suicide than for other MVs. The associations between ΔT and suicide were more significant in Sydney, Melbourne, Brisbane and Hobart than other cities. A higher UER was also associated with a higher suicide rate in Sydney, Melbourne, Brisbane and Perth. There was some interaction of ΔT on the association between UER and suicide in various cities.

### Temperature and suicide

In general, ΔT (in particular, temperature increases from last one month) had more significant impacts on suicide rates compared to other MVs included in analyses (e.g., temperature, rainfall and sunshine) in this study. These was a more significant association between ΔT and suicide among males than that in females in Sydney, Melbourne, Brisbane and all cities together. For ΔT, a higher RR was observed in violent suicide than that in non-violent suicide. This is consistent with previous studies [8, 13, 30]. The proposed mechanism for this association relates to lower levels in the action of serotonin 5-HT2A receptors in human body in winter, and increases with higher daily sunshine hours and temperature, especially in spring [31]. The receptors in human brain may have rapid responsiveness in the circumstance with increased air temperature [32]. Human mood may be influenced by the variation of serotonin 5-HT2A receptor activity. Thus suicidal behavior may be triggered during extreme weather, e.g., heatwave [33, 34]. In this study, there were marked increases in the suicide rate from winter to spring, particularly in Melbourne and Brisbane. In above cities (all in temperate climate zones), the temperature change was greater in relative magnitude from winter to spring than that from spring to summer. Thus higher ΔT may lead to higher suicide risks. However, Darwin has the highest mean temperature and lowest changes of temperature between months among the Australian capital cities. This may explain that ΔT in Darwin has a weaker association with suicide rate than in other cities. In general, the seasonality of suicide was associated with temperature change over months, and seasonality was demonstrated through different variables significantly associated with suicide (e.g., ΔT in Sydney and mean temperature in Darwin).

### Rainfall and suicide

There was no significant association between rainfall and suicide in Sydney in this study, which differs from previous NSW studies [22, 23]. Each of the studies covered both urban (including Sydney area) and rural areas. Usually prolonged droughts in rural and remote areas lead to decreased agricultural production and the income of the local population, especially farmers [35–38]. Thus mental health problems, such as anxiety, despair, may emerge in drought affected areas, where the availability of mental health care and other social services is more limited compared to urban areas and a potential driver of suicidal behavior in these areas [39]. However, the socioeconomic impact of drought in urban areas may be not as dramatic as that in rural areas. So it is difficult to find the association between rainfall and suicide rate in most urban areas, including Sydney. There was an increased suicide risk in months with less rainfall in Melbourne, when temperature was also high in these months. No significant effects of rainfall were found in other cities.

### Sunshine and suicide

The significant association between sunshine and suicide rate was only found among males in Melbourne in this study, which is consistent with the previous studies in Victoria (including Melbourne area) and other places [10, 40–43]. The study by Vyssoki et al. in Austria also indicated a positive short term (lag of ten days and less) effect but negative mid-long term (lag of 14–60 days) effect of sunshine on daily suicide [40]. Our study used monthly data and examined the association of sunshine and suicide in the same month, thus the lag effect could not be detected. Some studies measured more specific parameters of serotonin metabolism, suggesting that extracellular serotonin is low during winter, especially with less sunshine, which may have a protective effect on human mood [31, 41]. A higher level of serotonin in summer can trigger impulsiveness and aggression, which may lead to some violent self-harm behaviours [31, 34, 36, 41]. Melbourne lies on high latitude location in Australia with less rainfall in summer than that in winter. Thus sunshine peaks in summer (December and January) and drops to bottom in July (winter), and sunshine change is positively correlated with ΔT. However, Darwin, a tropical city, has plenty of rainfall in wet season (summer in other cities) and rare in dry season (winter in other cities); then there is less sunshine in wet season than in dry season and sunshine is negatively correlated with temperature. This may explain the opposite trends of the associations of suicide rate with rainfall and sunshine and in Darwin (although not significant) and in Melbourne.

### Unemployment and suicide

In this study, higher UER were significantly associated with suicide in most of the Australian capital cities, even after adjusting for seasonality and for lagged periods of 1 to 6 months). This is consistent with other Australian and international studies [44–46]. Usually, unemployment can lead to the reduction of personal and family income, add the stress, tension between family members and despair, especially for long term unemployment with limited financial aid from government and civil organizations [44–46]. Thus suicidal behaviors may be resulted from deteriorating economic conditions. This study also tested other lag effect of 2 to 6 months between unemployment and suicide and found that the trend was as similar as 1 month lag.

### The interaction of ΔT and unemployment with suicide

Both of socioeconomic variables and physical response to climate factors can influence human mood and were associated with suicide seasonality [47]. Thus the interaction of ΔT and UER with suicide may significantly associated with suicide, especially during a period with both of high UER and dramatic temperature change over time (ΔT) among a relatively large size of population and suicide cases in particular area [48]. This finding was consistent for Sydney, Melbourne and Brisbane. The three cities represent approximately 7 % of all capital city populations and 73 % of suicide cases. Thus it can explain the similarity of the findings in each of the three cities as those in capital cities altogether, compared to other capital cities having much smaller population and suicide number. In general, most of variables were more significantly associated with male suicide than female suicide. However, the interaction analysis indicated that female suicide had higher RR than male suicide (e.g., Sydney, Brisbane and all eight cities together), suggesting that females were more vulnerable in some circumstances, particularly in months when both UER and ΔT were high during the study period.

### Strengths and limitations

This study has three key strengths. Firstly, this is the first study exploring the association between MVs and suicide in major Australian cities over time. Secondly, both MVs and UER were taken into account, and the key risk factors at a macro level were investigated in different cities. This study also examined the interactive effect of MVs and a key socio-economic determinant of suicide, and investigated the extent to which the epidemiology of suicide in Australia differs by geographically and meteorologically distinct areas. Finally, the findings in this study may help public health decision-makers and practitioners to improve current suicide control and prevention strategies.

However, some limitations should also be acknowledged. Firstly, more detailed personal information of each suicide case, e.g., health status and mental disorder before suicide, consumption of alcohol at the individual level, economic condition, intake of omega-3 fatty-acid, were not available in the dataset. Secondly, using monthly meteorological data may mask some extreme weather conditions, e.g., short term heat waves, which may have potential impact on mental health among population. Finally, the impact of climate and unemployment on suicide after 2005 was not examined due to availability of dataset.

## Conclusion

In general, the associations between MVs and suicide rates differed across Australian capital cities. As some non-capital urban areas, rural and remote areas also have a high risk of suicide, it is necessary to explore the potential socio-environmental determinants of suicide using comprehensive national data, especially from spatiotemporal aspects. Suicide control and prevention strategies can be more targeted and specific on the basis of better understanding of the geographical and meteorological patterning of key socio-environmental risk factors associated with suicide.
